# Effects of free weight and body mass‐based resistance training on thigh muscle size, strength and intramuscular fat in healthy young and middle‐aged individuals

**DOI:** 10.1113/EP090655

**Published:** 2023-05-03

**Authors:** Madoka Ogawa, Yuto Hashimoto, Yukina Mochizuki, Takamichi Inoguchi, Ayumu Kouzuma, Minoru Deguchi, Mika Saito, Hiroki Homma, Naoki Kikuchi, Takanobu Okamoto

**Affiliations:** ^1^ Faculty of Sociology Kyoto Sangyo University, Motoyama, Kamigamo, Kita‐ku Kyoto Japan; ^2^ Faculty of Sport Science Nippon Sport Science University Tokyo Japan; ^3^ Graduate School of Health and Sport Science Nippon Sport Science University Tokyo Japan

**Keywords:** adipose tissue, hypertrophy, magnetic resonance imaging, strength training

## Abstract

The objective of this study was to investigate the effects of free weight and body mass‐based resistance training (RT) on muscle size and thigh intramuscular fat (IMF) in young and middle‐aged individuals. Healthy individuals (aged 30–64 years) were assigned to either a free weight RT group (*n* = 21) or a body mass‐based RT group (*n* = 16). Both groups performed whole‐body resistance exercise twice a week for 8 weeks. Free weight resistance exercises (squats, bench press, deadlift, dumbbell rows and back range) involved 70% one repetition maximum, with three sets of 8–12 repetitions per exercise. The nine body mass‐based resistance exercises (leg raise, squats, rear raise, overhead shoulder mobility exercise, rowing, dips, lunge, single‐leg Romanian deadlifts and push‐ups) included the maximum possible repetitions per session, which were performed in one or two sets. Mid‐thigh magnetic resonance images using the two‐point Dixon method were taken pre‐ and post‐training. The muscle cross‐sectional area (CSA) and IMF content in the quadriceps femoris were measured from the images. Both the groups showed significantly increased muscle CSA post‐training (free weight RT group, *P* = 0.001; body mass‐based RT group, *P* = 0.002). IMF content in the body mass‐based RT group significantly decreased (*P* = 0.036) but did not significantly change in the free weight RT group (*P* = 0.076). These results suggest that the free weight and body mass‐based RTs could induce muscle hypertrophy; however, in healthy young and middle‐aged individuals, decreased IMF content was induced following the body mass‐based RT alone.

## Abstract (highlights)

- **What is the central question of this study?**
  How do free weight resistance training (RT) and body mass‐based RT for 8 weeks compare for isometric muscular strength, muscle size and intramuscular fat (IMF) content in the quadriceps femoris?
- **What is the main finding and its importance?**
  Free weight and body mass‐based RTs could induce muscle hypertrophy; however, decreased IMF content was observed following the body mass‐based RT alone.

## 1 INTRODUCTION

Adipose tissue/fat distribution plays an important role in metabolic and cardiovascular disease development (Koenen et al., 2021), chronic inflammation (Rana & Neeland, 2022), impaired glucose tolerance (Tilves et al., 2020), lower muscular strength (Goodpaster, Carlson et al., 2001) and functional performance (Buford et al., 2012; Goodpaster, Carlson et al., 2001; Hilton et al., 2008; Tuttle et al., 2012). An accumulation in ectopic adipose tissue or fat within or between muscle fibres is called ‘intramuscular fat(IMF), and its role has been studied extensively. IMF accumulation develops in response to various pathological conditions. These conditions are the result of ageing (Akima et al., 2015), decreased physical activity levels (Manini et al., 2007), spinal cord injury (Gorgey & Dudley, 2007), obesity and type 2 diabetes (Yu et al., 2021). Magnetic resonance imaging (MRI) is the gold standard for assessing human skeletal muscle quality and quantity and investigating the IMF content of targeted muscles, and has been frequently used in previous studies (Hogrel et al., 2015; Ogawa et al., 2020).

Previous studies consistently found a common result: increased adipose tissue or fat within or between muscle fibres and fascia associated with lower muscular strength (Goodpaster, Carlson et al., 2001; Hilton et al., 2008). These findings imply that lower IMF is vital for muscular strength maintenance in addition to muscle size, thereby necessitating effective methods for decreasing IMF. A previous study reported that resistance exercise causes muscle hypertrophy, which also decreases the total intermuscular adipose tissue's cross‐sectional area (CSA) within the fascia‐included IMF (Marcus et al., 2010). However, a recent study showed that 8 weeks of high‐load free weight resistance training (RT) did not considerably change the IMF content in healthy young individuals (Bolsterlee et al., 2021). These findings indicate that even if free weight RT reduces the IMF content, the effect is small in young individuals without obesity or metabolic dysfunction.

Body mass‐based resistance exercise (e.g., squats), which can be performed by everyone anywhere, are feasible and effective for improving knee extensor muscular strength in older adults (Yoshiko & Watanabe, 2021) and body fat percentage and knee extensor muscular strength in adolescents (Takai et al., 2013). Interestingly, in young men, body mass‐based squat exercises involve aerobic metabolism after 5 min from exercise onset (Haramura et al., 2017). This indicates that body mass‐based resistance exercise would be predominately supported by aerobic metabolism when continuously performed. Therefore, the body mass‐based RT may reduce the IMF content more than the free weight RT.

In the present study, we compared the effects of free weight RT and body mass‐based RT on muscle size and IMF content in healthy young and middle‐aged individuals. We hypothesised that body mass‐based RT induces muscle hypertrophy and improves muscular strength to a similar extent to free weight RT, as well as reducing IMF content to a larger extent than free weight RT.

## 2 METHODS

### 2.1 Ethical approval

Prior to study initiation, all participants provided written informed consent, and the study was approved by the ethics committee of Nippon Sport Science University (020‐G04). The study was performed in accordance with the *Declaration of Helsinki*, 2013.

### 2.2 Participants

The present study was conducted as part of health promotion classes for volunteers at Nippon Sport Science University from 2019 to 2020. The participants learned about this class through a public relations website and advertisement, and they applied to participate in the class on their own accord. The present study was designed as a non‐randomised controlled trial with standardised training conditions within the class. Practical considerations required that all participants in each class perform the same exercises; therefore, participants enrolled during the first year (2019) were assigned to the free weight RT group, and those enrolled during the second year (2020) were assigned to body mass‐based RT. Different individuals attended the class during the first and second years.

The inclusion criteria were as follows: (1) residence in Tokyo; (2) aged 30–64 years; (3) no conditions requiring exercise restriction (e.g., cardiac disease, respiratory disease, hypertension and orthopaedic conditions); (4) body mass index (BMI) < 30.0 kg/m^2^; and (5) reporting no involvement in regular physical activity for at least 1 year prior to the intervention period. Overall, 42 healthy men and women were eligible to participate in the present study. Five participants failed to complete the 8‐week intervention and measurements; however, none of the participants dropped out because of injury or illness. Of the 37 participants who completed the study, 21 were assigned to the free weight RT group (six men and 15 women) and 16 was assigned to the body mass‐based RT group (six men and 10 women).

### 2.3 Training protocols

**FIGURE 1:**
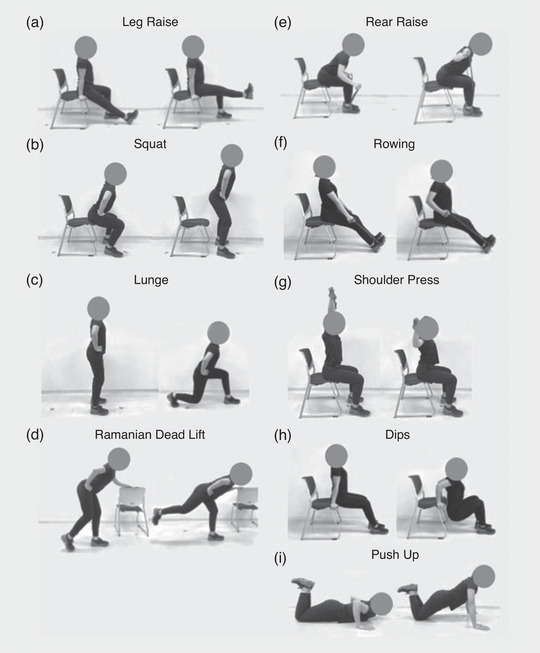
Exercise protocol cited by Kikuchi et al. (2021): (a) leg raise, (b) squat, (c) lunge, (d) single‐leg Romanian deadlift, (e) rear raise, (f) rowing, (g) overhead shoulder mobility exercise (shoulder press), (h) dips, (i) push‐up.

The body mass‐based RT group performed exercises twice a week for 8 weeks for a total of 16 sessions, with each session lasting for 60 min. The sessions included nine body mass‐based resistance exercises (leg raise, squat, rear raise, overhead shoulder mobility exercise, rowing, dips, lunge, single‐leg Romanian deadlift and push‐ups). Prior to the exercise programme, the participants attended a training class on each exercise. Under the supervision of the National Strength and Conditioning Association Certified Strength and Conditioning Specialists (NSCA‐CSCS), the participants were instructed to exceed the number of repetitions and sets shown in Table 1. All participants performed as many repetitions as possible for each session. The body mass‐based RT programmes were described in detail in our previous study (Kikuchi et al., 2021). The subjects performed RT while standing, sitting on a chair or using an elastic band (Figure 1). Rear raise, overhead shoulder mobility exercise and rowing were performed using an elastic band.

**TABLE 1:** The body mass‐based resistance exercises and the number of repetitions and sets.

	1–4 weeks		5–8 weeks	
	Number of repetitions	Set	Number of repetitions	Set
Leg raise	10–15	1	10–15	2
Squats	10	2	15	2
Overhead shoulder mobility exercise	10–15	2	10–15	2
Rear raise	10–15	2	10–15	2
Rowing	10–15	2	10–15	2
Dips	10	2	10	2
Rumanian dead lift	8–10	1–2	8–10	2
Lunge	10–12	1	10–12	1
Push‐up	5–10	1–2	10–15	2

The free weight RT group performed exercises twice a week for 8 weeks for a total of 16 sessions, which included five exercises (squat, bench press, deadlift, dumbbell row and back range), performing 70% one repetition maximum (1RM), three sets of 8−12 repetitions per exercise. The subjects performed the 1RM test using free weight under the supervision of NSCA‐CSCS before the training period. The participants continuously performed the exercise until exhaustion. From the third training session, the force level was increased by 5% in each session until the participant could only perform eight repetitions. In subsequent sessions, if the participant improved in such a way that they could perform more than 10 repetitions in two adjoining sessions, the force level was again increased by 5%. If a participant could not successfully complete six repetitions in two successive sessions, the force level was subsequently decreased by 5%.

The training participation rate was 91.4% for the body mass‐based RT group and 93.8% for the free weight RT group, and there were no statistically significant differences between the groups. All training routines were directly supervised by the research team to ensure proper performance. To avoid potential dietary factors confounding the results, the participants were advised to maintain their customary nutritional regimen during the study.

### 2.4 Measurements

#### 2.4.1 Knee extension maximum voluntary contraction torque

The knee extension maximum voluntary contraction (MVC) torque was determined using a Biodex System 3 dynamometer (Biodex Medical Systems, Shirley, NY, USA). The participants were seated on a chair with their hip joint angle positioned at 90°. The knee joint's centre of rotation was visually aligned with the axis of the dynamometer's lever arm. The right ankle was firmly attached to the dynamometer's lever arm using a strap. After a warm‐up consisting of submaximal contractions, the participants were instructed to perform maximal isometric knee extensions at a knee joint angle of 90°. A knee joint angle of 0° corresponded to full extension of the knee. Each extension was held for 4 s. Warm‐ups were performed at 30%, 50% and 80% of MVC, with rests of 1 min between sets. The sampling rate were 1 kHz. The MVC was measured two times, and the interval between MVCs was 3 min. The coefficient of variation and reproducibility were <5%.

#### 2.4.2 Magnetic resonance imaging

**FIGURE 2:**
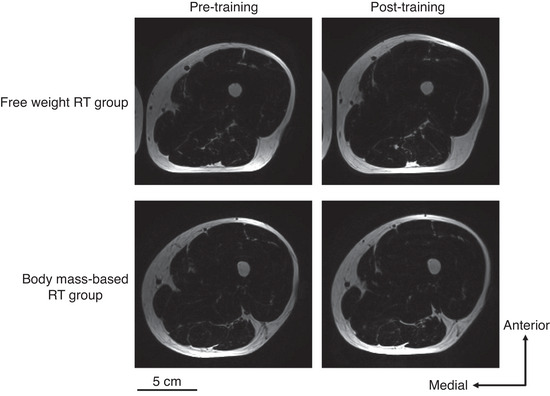
Typical fat image examples in pre‐ and post‐training for the free weight resistance training (RT) group and the body mass‐based RT group.

**FIGURE 3:**
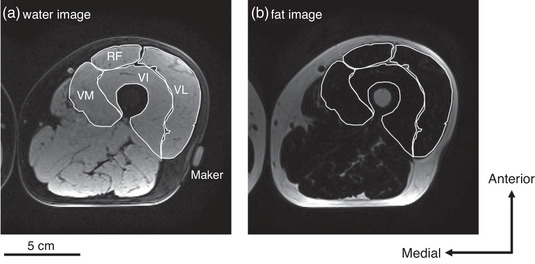
Typical magnetic resonance image examples using fat‐water separated two‐point Dixon imaging: (a) water image and (b) fat image. RF, rectus femoris; VI, vastus intermedius; VL, vastus lateralis; VM, vastus medialis.

Magnetic resonance (MR) scans were performed pre‐ and post‐training in both groups (Figure 2). The participants lay at rest for more than 5 min in the scan room. The measurements were made at intervals of 48–96 h after the final training session. The participants were assessed using a 1.5 T whole‐body MRI scanner (Echelon Oval; Hitachi, Ltd, Tokyo, Japan). After placing the participants in a supine position, thigh images were acquired using a torso coil. The mid‐thigh was defined according to markers attached to the middle point between the greater trochanter and the lateral condyle of the femur (Figure 3a). Two‐point Dixon transaxial images of the right thigh were obtained using the following sequence parameters: three‐dimensional; repetition time, 13.3 ms; echo times, 6.7 and 9.2 ms; flip angle, 60°; optimised field of view, 256 × 256 mm; slice thickness, 5 mm; and interslice gap, 0 mm. All participants were instructed to remain as motionless as possible.

#### 2.4.3 Thigh muscle composition analysis

We analysed the fat and water images using ImageJ software (v.1.44; National Institutes of Health, Bethesda, MD, USA) and used serial axial images to identify muscle boundaries. We then calculated the CSA of four muscles (rectus femoris, vastus lateralis, vastus intermedius and vastus medialis) at the mid‐thigh using water image (Figure 3a). The sum of the CSAs of the four muscles was then calculated as the CSA for the quadriceps femoris (QF). Furthermore, ImageJ software was used to measure the mean signal intensities from the water and fat images (Figure 3a, b) in the four muscles. We quantitatively assessed the fat fraction, which is the fat signal divided by the sum of the fat and water signals. The fat fraction for each muscle was calculated using the following equation (Dixon, 1984):

$$\begin{array}{ccc} \mathrm{Fat}\;\mathrm{fraction}\;(\%) & = & 100\times \mathrm{mean}\;\mathrm{fat}\;\mathrm{intensity}/(\mathrm{mean}\;\mathrm{water}\;\mathrm{intensity}\\ & & +\;\mathrm{mean}\;\mathrm{fat}\;\mathrm{intensity}) \end{array}$$

We calculated the mean fat fraction of the four muscles as the IMF content in the QF.

All images were analysed in random order by one of the researchers (M.O.). To confirm the reproducibility, we analysed the same MR image twice from 10 randomly selected participants for muscle CSA and IMF content of the QF. The intraclass correlation coefficients for muscle CSA and IMF content of the QF at the mid‐thigh were 0.97 and 0.98, respectively (both *P* < 0.001).

#### 2.4.4 Body composition assessment

Height was measured using a stadiometer with participants in a standing position with their shoes removed, shoulders relaxed, facing forward and the back toward the wall. With the participants wearing minimal clothing, we measured their body weight and body fat percentage using bioelectrical impedance (InBody Co. Ltd, Seoul, South Korea) with hand grip and foot plate electrodes. BMI was calculated as weight divided by height squared (kg/m^2^).

### 2.5 Statistical analysis

Prior to analysis, all variables were checked for outliers via stem and leaf plots. The Gaussian distribution for the measurements were tested using the Shapiro–Wilk test. Group differences at baseline were tested using Student's independent *t*‐test. Wilcoxon's signed‐rank test was performed for comparison pre‐ and post‐training in each group. The Aspin–Welch *t*‐test was used to determine the percentage changes pre‐ and post‐training in the knee extension MVC torque and muscle CSA, and the delta on IMF content for the comparison between the free weight RT group and the body mass‐based RT group. The relationship between the baseline and percentage change or delta using Spearman's correlation were tested in each group. The level of significance was set at 5%. All statistical analyses were performed using IBM SPSS Statistics v.25 (IBM Japan, Ltd, Tokyo, Japan).

## 3 RESULTS

### 3.1 Baseline measurements

The baseline measurements for the free weight RT group and body mass‐based RT group are shown in Table 2. The MVC torque and muscle CSA for the RT group were significantly higher than those for the body mass‐based RT group (MVC torque *P* = 0.014, muscle CSA *P* < 0.001); however, there were no statistically significant differences in age (*P* = 0.156), height (*P* = 0.294), body weight (*P* = 0.639), BMI (*P* = 0.774), body fat percentage (*P* = 0.108) and IMF content (*P* = 0.705).

**TABLE 2:** The baseline measurements in the free weight RT group and body mass‐based RT group.

	Free‐weight RT group	Body mass‐based RT group	*P*
Men/women	6/15	6/10	—
Age (years)	50.6 (6.8)	45.8 (11.1)	0.156
Height (cm)	161.3 (6.3)	164.4 (8.3)	0.294
Body weight (kg)	58.7 (9.4)	59.3 (9.0)	0.639
BMI (kg/m^2^)	22.2 (2.7)	21.9 (2.2)	0.774
Body fat (%)	26.7 (6.8)	23.0 (6.5)	0.108
MVC torque (Nm)	213.0 (61.9)	157.2 (40.7)	0.004
Muscle CSA (cm^2^)	73.5 (15.5)	53.3 (8.9)	<0.001
IMF content (%)	9.2 (2.8)	9.3 (5.0)	0.705

Data are shown as means (SD). Abbreviations: BMI, body mass index; CSA, cross‐sectional area; IMF intramuscular fat; MVC, maximum voluntary contraction; RT, resistance training.

There were no significant changes in body weight, BMI and body fat percentage between pre‐training and post‐training in either group (Table 3).

**TABLE 3:** Body weight, BMI and body fat percentage changes in the free weight RT group and body mass‐based RT group.

Variable	Free weight RT group						Body mass‐based RT group					
	Pre‐training		Post‐training				Pre‐training		Post‐training			
	Mean (SD)	Median (IQR)	Mean (SD)	Median (IQR)	*P*	ES (*r*)	Mean (SD)	Median (IQR)	Mean (SD)	Median (IQR)	*P*	ES (r)
Weight (kg)	58.7 (9.4)	56.4 (50.0–65.5)	58.5 (9.5)	57.0 (49.5–64.2)	0.87	0.00	59.3 (9.0)	57.4 (51.3–67.7)	59.6 (9.1)	57.0 (52.6–67.9)	0.90	−0.13
BMI (kg/m^2^)	22.2 (2.7)	22.1 (19.9–25.3)	22.3 (2.7)	21.4 (20.2–24.3)	0.99	0.00	21.9 (2.2)	21.6 (19.8–23.4)	21.9 (2.2)	21.6 (19.8–23.7)	0.72	0.00
Body fat (%)	26.7 (6.8)	28.5 (20.8–31.9)	26.2 (6.5)	28.2 (22.7–31.1)	0.09	−0.38	23.0 (6.5)	21.6 (17.1–27.0)	22.8 (6.5)	21.1 (17.3–27.8)	0.40	−0.20

Data are shown as means (SD). Abbreviations: BMI, body mass index; ES, effect size; IQR, interquartile range; RT, resistance training.

### 3.2 Training effects on MVC torque

**FIGURE 4:**
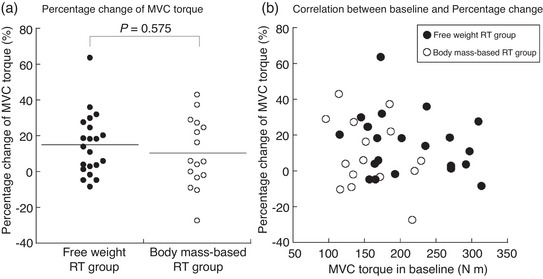
(a) The percentage change in knee extension MVC torque between the free weight RT group and the body mass‐based RT group. (b) Relationship between baseline knee extension MVC torque and the percentage change in MVC torque in the free weight RT group and the body mass‐based RT group. MVC, maximum voluntary contraction; RT, resistance training.

No significant changes were observed in knee extension MVC torque in the body mass‐based RT group between pre‐ and post‐training (*P* = 0.078, Table 4). Conversely, the MVC torque in the free weight RT group was significantly increased post‐training (*P* = 0.001, Table 4). Figure 4a shows the percentage change on the MVC torque between the groups. The MVC torque percentage change was not significantly different between the groups (*P* = 0.575, Figure 4a). There was no significant relationship between the baseline MVC torque and percentage change in the MVC torque pre‐ and post‐training (Figure 4b; free weight RT group, *r*_s_ = −0.197, *P* = 0.391; body mass‐based RT group, *r*_s_ = −0.174, *P* = 0.520).

**TABLE 4:** Mean values for the knee extension MVC torque and the QF CSA and IMF content in the free weight RT group and body mass‐based RT group.

Variable	Free weight RT group						Body mass‐based RT group					
	Pre‐training		Post‐training				Pre‐training		Post‐training			
	Mean (SD)	Median (IQR)	Mean (SD)	Median (IQR)	*P*	ES (*r*)	Mean (SD)	Median (IQR)	Mean (SD)	Median (IQR)	*P*	ES (*r*)
MVC torque (Nm)	213.0 (61.9)	192.4 (164.7–271.1)	242.5 (70.5)	238.0 (184.0–294.4)	<0.01	−0.74	157.2 (40.7)	150.0 (125.3–186.5)	171.0 (45.7)	164.5 (128.8–213.3)	0.08	−0.43
Muscle CSA (cm^2^)	73.5 (15.5)	68.4 (60.2–82.7)	78.1 (17.3)	75.1 (63.7–94.3)	<0.01	−0.75	53.3 (8.9)	51.5 (46.3–60.5)	55.2 (9.3)	53.5 (48.1–63.3)	<0.01	−0.75
IMF content (%)	9.2 (2.8)	8.8 (7.4–11.0)	8.4 (2.3)	7.7 (7.2–9.1)	0.08	−0.40	9.3 (5.0)	8.6 (5.3–12.7)	6.7 (2.6)	6.1 (5.3–8.2)	0.04	−0.51

Abbreviations: CSA, cross‐sectional area; ES, effect size; IMF, intramuscular fat; IQR: interquartile range; MVC, maximum voluntary contraction; QF, quadriceps femoris; RT, resistance training.

### 3.3 Training effects on muscle CSA

**FIGURE 5:**
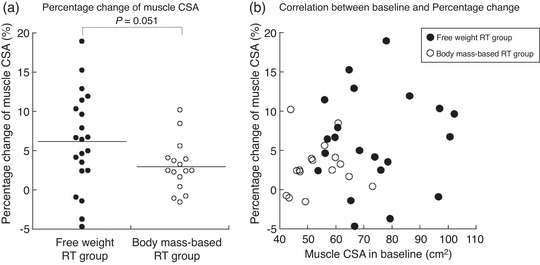
(a) The percentage change in muscle CSA in quadriceps femoris between the free weight RT group and the body mass‐based RT group. (b) Relationship between baseline muscle CSA in quadriceps femoris and the percentage change in muscle CSA in the free weight RT group and the body mass‐based RT group. CSA, cross‐sectional area; RT, resistance training.

The muscle CSA was significantly increased post‐training in both groups (free weight RT group, *P* = 0.001; body mass‐based RT group, *P* = 0.002, Table 4). The percentage change in the muscle CSA was not significantly different between the groups (*P* = 0.051, Figure 5a). There was no significant relationship between baseline muscle CSA and percentage change in muscle CSA pre‐ and post‐training in either group (Figure 5b; free weight RT group, *r*_s_ = 0.068, *P* = 0.769; body mass‐based RT group, *r*_s_ = 0.017, *P* = 0.949).

### 3.4 Training effects on IMF content

**FIGURE 6:**
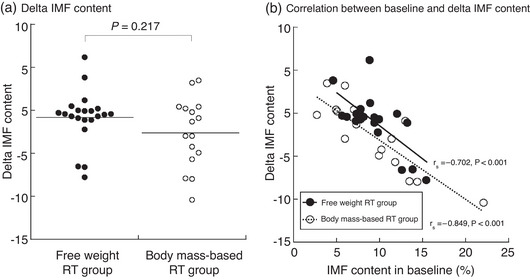
(a) The delta IMF content between the free weight RT group and the body mass‐based RT group. (b) Relationship between baseline IMF content in quadriceps femoris and the delta IMF content in the free weight RT group and the body mass‐based RT group. IMF, intramuscular fat; RT, resistance training.

There were no significant changes in the IMF content of the free weight RT group (*P* = 0.076), but in the body mass‐based RT group, there was a significant decrease post‐training (*P* = 0.036, Table 4). The delta IMF content pre‐ and post‐training was not significantly different between the groups (*P* = 0.217, Figure 6a). Furthermore, the baseline IMF content was significantly negatively correlated with the delta IMF content pre‐ and post‐training in both groups (Figure 6b; free weight RT group, *r*_s_ = −0.702, *P* < 0.001; body mass‐based RT group, *r*_s_ = −0.849, *P* < 0.001).

## 4 DISCUSSION

We investigated the effects of free weight RT and body mass‐based RT for 8 weeks on isometric muscular strength, muscle size and IMF content in healthy young and middle‐aged individuals. The results indicated that 8 weeks of free weight RT, but not body mass‐based RT, provided an adequate stimulus to promote isometric muscular strength gains in the knee extensor. Second, the muscle hypertrophy occurred in the both RTs, and rate was higher in free weight RT than in body mass‐based RT. Third, the body mass‐based RT group exhibited decreased IMF content, but the free weight RT group did not. Furthermore, the delta IMF content pre‐ and post‐training was significantly negatively correlated with the baseline in both groups. These results suggested that for isometric muscular strength gain and muscle hypertrophy, free weight RT is more effective, but body mass‐based RT could be a useful way to reduce IMF content. In addition, changes in the IMF content due to RT are also influenced by the baseline in healthy young and middle‐aged individuals.

The MVC torque in the free weight RT group was significantly increased post‐training but did not significantly change in the body mass‐based RT group in the present study (Table 4). However, muscle CSA was significantly increased post‐training in both groups (Table 4). Schoenfeld et al. (2017), with meta‐analyses, reported that muscle hypertrophy improvements are load‐independent, and an increase in muscular strength is superior in high‐load RT (>60% 1RM) compared with low‐load RT (≤60% 1RM). Previous studies reported that for young men, the vastus lateralis activity level during body mass‐based parallel squats exercise was 33% of that observed during MVC (Isear et al. 1997). Therefore, the body mass‐based RT used in the present study is included in the low‐load resistance exercise, which induces muscle hypertrophy and slightly affects muscular strength in healthy young and middle‐aged individuals consistent with Schoenfeld et al. (2017). Furthermore, previous studies reported a potential fibre type‐specific loading zone effect, with higher loads showing a greater increase in type II muscle fibre CSA and lower loads showing a greater increase in type I muscle fibre growth (Netreba et al., 2013). Considering our results together with the previous study result (Netreba et al., 2013), the body mass‐based RT might mainly relate to type I muscle fibre hypertrophy. However, the effect of body mass‐based RT on muscle fibre CSA has not been reported and needs further study.

In this study, participants with a wide range of age (30−64 years) and who reported no involvement in regular physical activities for at least 1 year before the intervention period were included and investigated. There were differences at baseline for MVC torque and muscle CSA (Table 2). However, no correlations were found between baseline and percentage change for MVC torque and muscle CSA (Figures 4a and 5b). Based on these results, the baseline differences in the present study could not be associated with the training effect. Conversely, a previous study reported that the magnitude of strength gain induced by body mass‐based RT is associated with the relative load on the working muscles (Ozaki et al., 2017). Such training would be particularly effective for older individuals who have relatively low strength.

The IMF content was significantly decreased post‐training in the body mass‐based RT group but not in the free weight RT group (Table 4). To the best of our knowledge, the present study is the first to show that body mass‐based RT could decreased IMF content in healthy young and middle‐aged individuals. Haramura et al. (2017) showed that body mass‐based squats exercise is of moderate intensity and involves aerobic metabolism after 5 min from exercise onset in healthy young men (Haramura et al., 2017). From these previous studies, body mass‐based RT was expected to have aerobic training aspects. A previous study of healthy young individuals demonstrated significant intramyocellular lipid (IMCL) utilization and its subsequent resynthesis during recovery from both endurance and resistance exercise (Loher et al., 2016). Furthermore, previous studies have shown that aerobic training significantly decreased IMCL in type 2 diabetes, whereas it increased in healthy individuals (Bajpeyi et al., 2012). Marcus et al. (2010) reported a mean relative decrease of 11% in CSA of intermuscular adipose tissue‐included IMF after 12 weeks of RT using eccentric exercise on the knee extensors, in which the muscle is lengthened as the participant attempts to slow down the external load being applied to the muscle by motorised ergometer movements (Marcus et al., 2010). The authors enrolled individuals older than 55 years who were cancer survivors, chronic stroke survivors, had impaired glucose tolerance, multiple sclerosis or had undergone total knee arthroplasty. The authors employed the RT intensity measured using the Borg scale, with a progression that proceeded from ‘very light’ to ‘somewhat difficult’ and a duration that lasted from 5 min to 30 min per day (Marcus et al., 2010). Although the characteristics of the participants and resistance exercises differ between the previous and present study, those findings suggested that body mass‐based RT could be a useful method for preventing IMF accumulation in healthy younger and middle‐aged individuals.

Interestingly, the IMF content did not significantly change between pre‐ and post‐training in the free weight RT group in the present study, which is consistent with a previous study (Bolsterlee et al., 2021) that demonstrated that high‐load RT affects IMF content as evaluated by the Dixon method. Bolsterlee et al. (2021) reported that 8 weeks of high‐load RT (three times per week, 80% 1RM, four sets of 10 repetitions) did not considerably alter the IMF content in young individuals (pre‐training 5.8 (1.9)%, post‐training 5.5 (1.3)%). Therefore, the results of the present study and Bolsterlee et al. (2021) were consistent in suggesting that free weight RT with over 70% 1RM does not appreciably change the IMF content when evaluated using the Dixon method in healthy young and middle‐aged individuals without metabolic deficiency or disease and a BMI < 30.0 kg/m^2^.

Furthermore, different MRI methods for assessing the IMF content could be related to the RT difference effects for IMF content between the previous studies (Bolsterlee et al., 2021; Marcus et al., 2010). Fischer et al. (2014) reported that IMF content determined via the two‐point Dixon method was closely related to the total IMCL and extramyocellular lipids (EMCL) detected by proton magnetic resonance spectroscopy (^1^H‐MRS) (*r* = 0.918–0.990, *P* < 0.001) (Fischer et al., 2014). Conversely, Akima et al. (2016) reported that the IMF content in the vastus lateralis calculated by T1‐weighted imaging was correlated with EMCL (*r* = 0.506, *P* < 0.01) but not IMCL (*r* = 0.263, n.s.). Therefore, IMF content assessed by the Dixon method in the present study could reflect both lipids inside and outside of the muscle cells. In the present study, one subject (a woman, 54 years old, BMI 19.6 kg/m^2^, body fat percentage 25.0%) in the body mass‐based RT group whose IMF content at post‐training was more than thrice that of the median was excluded as an outlier. This subject's pre‐training IMF content was 8.1% and post‐training was 24.1%. Along with this subject, there were two other subjects in the free weight RT group and two in the body mass‐based RT group who showed ≥3% post‐training IMF content than at pre‐training. The pre‐training IMF content of these four subjects (average 5.8%) was lower than the overall average. There were individual differences in the IMF content change in the present study, and the reason for these differences is unclear. However, the baseline IMF content was strongly associated with the delta IMF content due to the RT in both groups (Figure 6b). If the IMF content measured by the Dixon method mainly reflects the IMCL levels, there were several factors that could explain the present results. As noted above, IMCL was reduced by aerobic training in type 2 diabetics (Bajpeyi et al., 2012). Paradoxically, endurance athletes have higher IMCL (Goodpaster, He et al., 2001b), and long‐sent results are consistent with the previous studies in which subjects such as type 2 diabetics with a high baseline IMCL had a greater loss of IMCL post‐training, and subjects with a lower baseline IMCL increased or did not have any IMCL changes post‐training. However, there is little evidence on the effects of RT. Ngo et al. (2012) reported that IMCL was not significantly altered after 14 weeks of RT (three times per week, 20 repetitions, three sets) in older men (Ngo et al., 2012). Conversely, 16 weeks of RT (three times per week, 50–80% 1RM, 8–15 repetitions, three sets) significantly decreased IMCL in older women who were the offspring of obese/overweight mothers (Bucci et al., 2016). Furthermore, previous studies in rats demonstrated that RT decreased IMCL in the soleus but not in the gastrocnemius muscles, suggesting that RT plays a role in IMCL levels that depends on the muscle fibre composition (Ribeiro et al., 2017). On the other hand, the RT effects on EMCL are not clearly understood. Future studies need to investigate the factors that contribute to individual differences in IMF, including IMCL and EMCL changes by RT.

The present study has some limitations. First, it was a non‐randomised design and did not have a control group because it was part of a health promotion class for volunteers. Previous studies have reported that age (Akima et al., 2015; Gallagher et al., 2005), BMI (Gallagher et al., 2005), muscle size (Akima et al., 2015), disease (e.g., diabetes; Dubé et al., 2006), spinal cord injury (Gorgey & Dudley, 2007) and physical activity levels (Ogawa et al., 2021) are associated with the baseline IMF content. Therefore, future randomised controlled studies need to consider these confounding factors while investigating the impact of RT on muscle size and IMF content. Second, the RT period in the present study is short at 8 weeks and the long‐term effects should be investigated further. Third, the MR device did not have ^1^H‐MRS. It is necessary to confirm whether the same changes in the IMF content by the Dixon method occurred in IMCL and EMCL, which will be assessed by MRS in future studies.

In conclusion, in healthy young and middle‐aged individuals, the performance of free weight and body mass‐based RT for 8 weeks could induce muscle hypertrophy, and IMF content was decreased following the body mass‐based RT alone.

## AUTHOR CONTRIBUTIONS

All authors contributed to the study conception and design. Material preparation, data collection and analysis were performed by Madoka Ogawa, Yuto Hashimoto, Yukina Mochizuki, Takamichi Inoguchi, Ayumu Kouzuma, Minoru Deguchi, Mika Saito, Hiroki Homma, Naoki Kikuchi and Takanobu Okamoto. All authors have read and approved the final version of this manuscript and agree to be accountable for all aspects of the work in ensuring that questions related to the accuracy or integrity of any part of the work are appropriately investigated and resolved. All persons designated as authors qualify for authorship, and all those who qualify for authorship are listed.

## CONFLICT OF INTEREST

The authors of this manuscript declare no relationships with any companies whose products or services might be related to the subject matter.

## Supporting information

Statistical Summary Document

## Data Availability

Data generated or analysed during the present study are not available due to the nature of this research including personal information [the ethics committee of Nippon Sport Science University (020‐G04)].
