# Supplementary material for: Effects of free weight and body mass‐based resistance training on thigh muscle size, strength and intramuscular fat in healthy young and middle‐aged individuals
Source: Exp Physiol. 2023 May 3;108(7):975–85. doi: 10.1113/EP090655 (PMC10988481; doi:10.1113/EP090655)
Supplement: Supplementary file 1 — Statistical Summary Document [file EPH-108-975-s001.pdf]

Experimental Physiology

Statistical Summary Document

Manuscript Title: Effects of free weight and body mass-based resistance training on thigh muscle size and intramuscular fat in healthy young and middle-aged individuals

Authors: Madoka Ogawa, Yuto Hashimoto, Yukina Mochizuki, Takamichi Inoguchi, Ayumu Kouzuma, Minoru Deguchi, Mika Saito, Hiroki Homma, Naoki Kikuchi and Takanobu Okamoto

Animal model used, if a No

Underlying hypothesis: 1. We hypothesised that free-weight RT and body mass-based resistance training (RT) induces (A)muscle hypertrophy and (B) improves muscular strength, and (C) reduces intramucular fat (IMF) content.
2. In comparison those percent change or delta, we hypothesised that body mass-based RT muscle hypertrophy and improves muscular strength to a similar extent as free-weight RT, as well as reduces IMF content to a larger extent than free-weight RT.
3. We confirmed whether there is a correlation between the value of the baseline and the percent change/delta to RT.

Definitions of 'n': A priori effect size (t tests - Means: Difference between two dependent means (matched pairs), tails = two, effect size =0.8, α err prob = 0.05, Power (1-β err prob) = 0.95) required number of subjects was n = 23.

Statistical summary table:

Experimental question number	Finding/ conclusion	Experimental location/variable	Mean value	Standard Deviation	n	Exact P value	Figure/table in which data are presented	Units	Data comparisons	Statistical test	Any other experimental factors	Comments
**Multiple tests of the same question are shown in different rows**		**e.g. cortex vs cerebellum or genotype**	**(or other summary statistic)**	**(SD)**	**(value)**	**If P is considered significant against a stated confidence limit, authors may indicate this in bold.The exact value must be stated unless P is less than 0.0001, in which case < is permitted.**			**e.g. WT vs KO**		**e.g. subjects' age or sex**	**e.g. observation**
1	(A)The muscle hypertrophy occurred in the both RT.<br>(B) Free-weight RT provided an adequate stimulus to promote muscular strength gains in the knee extensor, but not in body mass-based RT.<br>(C) The body mass-based RT group exhibited decreased IMF content, but not the free-weight RT group did not.	(A) Muscle cross sectional area (CSA) in quadriceps femoris<br>(B) Knee extension maximum voluntary contraction (MVC) torque<br>(C) IMF content in quadriceps femoris.	(A) Free-weight RT; pre-training 73.5 and post-training 78.1. Body mass-based RT; pre-training 53.3 and 55.2.<br>(B) Free-weight RT; pre-training 213.0 and post-training 242.5. Body mass-based RT; pre-training 157.2 and 171.0.<br>(C) Free-weight RT; pre-training 9.2 and post-training 8.4. Body mass-based RT; pre-training 9.3 and 6.7	(A) Free-weight RT; pre-training 15.5 and post-training 17.3. Body mass-based RT; pre-training 8.9 and 9.3.<br>(B) Free-weight RT; pre-training 61.9 and post-training 70.5. Body mass-based RT; pre-training 40.7 and 45.7.<br>(C) Free-weight RT; pre-training 2.8 and post-training 2.3. Body mass-based RT; pre-training 5.0 and 2.6	Free weight RT group; N=21<br>Body mass-based RT group; N=16	(A) Free-weight RT, ***P* = 0.0010**<br>Body mass-based RT, ***P* = 0.0020**<br>(B) Free-weight RT, ***P* = 0.0010**<br>Body mass-based RT, *P* = 0.0780<br>(C) Free-weight RT, *P* = 0.0760<br>Body mass-based RT, ***P* = 0.0360**	Table 4	(A)cm^2<br>(B)Nm<br>(C) %	Pre-trainig vs. post-training	Wilcoxon's signed-rank test	Body weight, body mass index (BMI) and body fat percentage	intervention
2	(A)The percent change in the muscle CSA was not significantly different between the groups.<br>(B)The MVC torque percent change was not significantly different between the groups.<br>(C) The delta IMF content pre- and post-training was not significantly different between the groups.	(A) Muscle CSA in quadriceps femoris<br>(B) Knee extension MVC torque<br>(C) IMF content in quadriceps femoris.	(A) 6.2 in free-weight RT and 3.0 in body mass-based RT.<br>(B) 14.9 in free-weight RT and 10.2 in body mass-based RT.<br>(C) -0.8 in free-weight RT and -2.6 in body mass-based RT.	(A) 6.1 in free-weight RT and 3.2 in body mass-based RT.<br>(B) 17.2 in free-weight RT and 19.2 in body mass-based RT.<br>(C) 3.1 in free-weight RT and 4.0 in body mass-based RT.	Free weight RT group; N=21<br>Body mass-based RT group; N=16	(A) *P* = 0.0510<br>(B) *P* = 0.5750<br>(C) *P* = 0.2170	(A) Figure 5A<br>(B) Figure 4A<br>(C) Figure 6A	(A) %<br>(B) %<br>(C) delta	Free weight RT group vs. Body mass-based RT group	The Aspin–Welch t-test	-	
3	(A)There was no significant relationship between baseline and percent change in muscle CSA in the both groups.<br>(B)There was no significant relationship between baseline and percent change in MVC torque in the both groups.<br>(3)The baseline IMF content was significantly negatively correlated with the delta IMF content pre- and post-training in both groups.	(A) Muscle CSA in quadriceps femoris<br>(B) Knee extension MVC torque<br>(C) IMF content in quadriceps femoris.	Please refer to 1 and 2 above	Please refer to 1 and 2 above	Free weight RT group; N=21<br>Body mass-based RT group; N=16	(A) Free-weight RT group, r_s = 0.068, *P* = 0.769, and body mass-based RT group, r_s = 0.017, *P* = 0.949<br>(B)Free-weight RT group, r_s = −0.197, *P* = 0.391, and body mass-based RT group, r_s = −0.174, *P* = 0.520<br>(C) Free-weight RT group; r_s = 0.702, ***P* = 0.0001**, body mass-based RT group; r_s = 0.849, ***P* = 0.0001**	(A) Figure 5B<br>(B) Figure 4B<br>(C) Figure 6B	(A) cm^2, %<br>(B) Nm, %<br>(C) %, delta	The relationship between the baseline and percent change or delta	Spearman correlation	-	
